# IMU-based segmental root mean square analysis of gait in individuals with cerebellar ataxia: a pilot cross-sectional study

**DOI:** 10.1038/s41598-025-20775-3

**Published:** 2025-10-22

**Authors:** Janice Mendonca, Abraham M. Joshua, Shashank Shetty, Krishnan Chemmangat, Shyam Krishnan, K. Vijaya Kumar, Zulkifli Misri, Rohit Pai, Shivananda Pai

**Affiliations:** 1 Department of Physiotherapy, Kasturba Medical College Mangalore, Manipal Academy of Higher Education, Manipal, India; 2 Department of Electrical and Electronics Engineering, National Institute of Technology Karnataka, Surathkal, India; 3 Department of Neurology, Kasturba Medical College Mangalore, Manipal Academy of Higher Education, Manipal, India

**Keywords:** Cerebellar ataxia, Gait ataxia, Gait analysis, Inertial sensors, Biotechnology, Neuroscience, Medical research, Neurology, Engineering

## Abstract

Cerebellar ataxia (CA) affects limb movement, balance, and gait. Subjective rating scales like Scale for the Assessment and Rating of Ataxia (SARA) may underestimate gait severity. Inertial measurement units (IMUs) offer an objective gait analysis. Impaired trunk control might compromise gait performance and stability in individuals with ataxia. This study quantified trunk kinematics and gait parameters using Root Mean Square (RMS) values, comparing CA to healthy individuals. Ten CA cases and twenty healthy controls were recruited. Six IMU sensors positioned at anatomical landmarks recorded data via two ESP32 microcontrollers using Wi-Fi. Participants walked a 10-meter path at a self-selected pace. RMS mean linear and angular velocity and angular deviation were calculated. Individuals with CA showed decreased mediolateral linear acceleration at the left shoulder (*p* = 0.001) and an increased vertical linear acceleration at the right ankle *(p = 0.015)*, left shoulder *(p = 0.028)*, and back (*p = 0.019*). Total angular velocity was lower at the right shoulder *(p = 0.017)*, left shoulder *(p = 0.005)*, back *(p = 0.002)*, and both ankles (right: *p* = 0.001; left: *p* = 0.001). The correlation between IMU-derived features and SARA-gait score in the CA group was not statistically significant (all *p* > 0.05), except for the right shoulder’s mediolateral angular velocity (*p = 0.046*). Both ankle segments’ angular deviations (right: *p* = 0.001; left: *p* = 0.006) were reduced. The CA group revealed reduced RMS linear and angular velocities. IMU-based trunk and gait analysis provides a more objective method that would help in planning targeted rehabilitation treatments.

Trial registration: The study was approved by the Institutional Ethics Committee (IEC), Kasturba Medical College, Mangalore, Manipal Academy of Higher Education (IEC KMC MLR 12/2023/483) on 21st December 2023 and the Clinical Trial Registration (CTRI/2024/07/070614) on July 15th, 2024.

## Introduction

Cerebellar ataxia (CA) represents a neurological movement disorder that impacts limb coordination, balance, gait, oculomotor control, and cognitive processes^1^. Various neurological conditions, both hereditary and acquired causes, such as spinocerebellar ataxia, cerebellar stroke, traumatic brain injury, and multiple system atrophy-cerebellar type, can lead to CA^2,3^. The most noticeable motor symptom of damage to the cerebellum or its intricate connections is gait ataxia^4^. This particular impairment is commonly described as “drunken gait”^5^. It is characterized by decreased walking pace, cadence, step length, stride length, and swing phase. Additionally, there is a notable increase in stance width, stride time, step time, double limb support phase, and an overall rise in walking variability concerning step length, stride length, and stride time^6,7^. Inter-limb incoordination, balance impairment, and incoordination during postural activities can all contribute to gait variability^1^.

Individuals with CA show deficits in intralimb coordination and multi-joint movement. Poor dynamic balance during walking is caused by improper foot placement, resulting from lower limb incoordination. The cause behind an individual overshooting a target is dysmetria, a delay in activating the antagonist muscle compared to the agonist muscles that accelerate the limbs. Dynamic balance while walking is impacted by the dysmetria and dyssynergia of the lower extremities^1,8^. Midline cerebellar lesions cause truncal ataxia. Individuals with CA may exhibit oscillations of the body when standing (titubation) or sitting as a sign of truncal instability^9^. Trunk displacement and velocity are elevated both in the anterior-posterior (AP) and mediolateral (ML) planes in individuals diagnosed with CA^8,10–12^.

The primary methods for assessing gait in a clinical setting involve subjective rating scales such as the Scale for the Assessment and Rating of Ataxia (SARA) and the International Cooperative Ataxia Rating Scale (ICARS)^6,13,14^. However, evidence suggests that clinical evaluation tools may understate the intensity of gait changes in ataxic individuals as they often rely on subjective judgments and clinician experience. This reliance can hinder their ability to deliver an objective gait assessment^8,15^. Instrumented gait analysis techniques, on the other hand, are quantitative methods designed to measure subtle characteristics that may not be detectable by clinical examination. Such techniques can be categorized into three categories: wearable sensors, floor-mounted sensors, and imaging sensors. Wearable sensors can capture a variety of data that can characterize human gait. These consist of inertial measurement units (IMUs), pressure sensors, active tracking markers, strain gauge sensors, goniometers, and electromyography^16^.

IMUs are body-worn sensors that are increasingly being employed in clinical settings to gather objective data for analyzing gait. IMU-based systems have several benefits, such as affordability, portability, user-friendliness, suitability for outdoor use, and a simple calibration process^17^. Accelerometers and gyroscopes are the most commonly used IMUs and can sense linear acceleration and angular velocity about one or several axes, respectively. These can be combined to construct a comprehensive inertial gait-sensing system^18,19^.

Currently, a range of algorithms has been developed to identify gait patterns using inertial data collected from various sensors placed on the feet, hands, sternum, and lumbar areas^20–23^. On the other hand, some studies have only shown the use of lumbar-level sensors^24–26^. The majority of research showed that using inertial sensors can effectively assess gait among ataxic individuals. Interestingly, the movement of the head and trunk was not considered in any of the above-mentioned studies.

Individuals with ataxia might display enlarged head and trunk movements, which may signal difficulties in controlling their upper bodies during locomotion. Such pronounced upper body oscillations shift the center of gravity (COG) towards the edges of the base of support (BOS), impacting certain aspects of gait and increasing body sway and instability^27^. Numerous studies underscore the significance of trunk movements in human gait and highlight that the coordination between upper and lower body segments is crucial for efficient gait^4,28^. Lack of coordination might play a critical role in impairing gait performance and stability in individuals with ataxia. Hence, conducting a comprehensive study based on trunk movements with respect to gait in individuals with CA using IMU is imperative^28^.

## Methods

### Participants

**Fig. 1:**
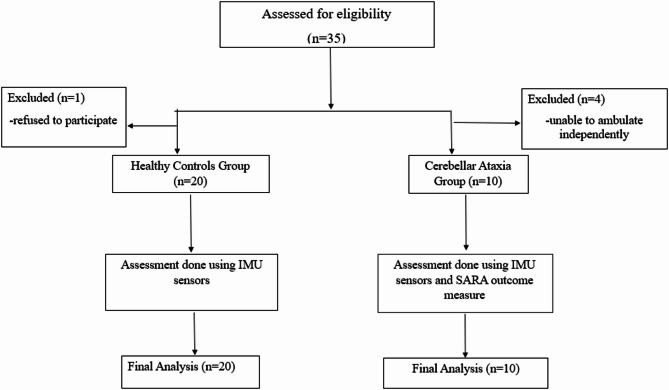
STROBE flow diagram of the participants selected.

This cross-sectional analytical pilot study enrolled 30 participants after the screening, of which 20 were healthy individuals, and 10 were individuals with CA (Fig. 1).

We included individuals diagnosed with CA who were able to walk independently without any walking aids. Healthy controls were individuals with no history of any neurological, musculoskeletal, or psychiatric illness or disease, no positive family history of neurodegenerative disease, and no obvious clinical signs upon examination suggestive of any neurological or other system disorders. Participants in both groups were within the age range of 18 to 65 years. As this was a pilot study aimed at assessing feasibility, no formal power analysis was performed.

Exclusion criteria included subjects with other neurological conditions, such as tumors, Parkinson’s disease, and individuals post-surgery, as well as those with severe visual or hearing disturbances, severe cognitive impairments, and orthopedic constraints affecting gait. The groups differed in sample size due to the constraints in recruitment specific to the CA population, its clinical rarity, and mobility impairments.

The protocol was submitted to the Scientific Committee and Institutional Ethics Committee (IEC), Kasturba Medical College, Mangalore, Manipal Academy of Higher Education. After the approval from the Institutional Ethics Committee (IEC KMC MLR 12/2023/483) and the completion of the Clinical Trial Registration (CTRI/2024/07/070614), the study commenced. The study was conducted in accordance with the ethical standards outlined in the Declaration of Helsinki. The consulting neurologists referred individuals diagnosed with CA to the study. Eligible participants were informed about the study, and written informed consent was obtained. Subsequently, subjects meeting the inclusion and exclusion criteria of the study were enrolled from August 1st, 2024. Demographic details were collected, and individuals with CA were assessed using the SARA outcome measure.

The mean age of the CA subjects was (50.8 ± 9.85) years, and the healthy participants were 48.4 ± 11.1 years. The majority were males in both groups, CA subjects (*n* = 9) and healthy individuals (*n* = 13), resulting in an unbalanced gender distribution. The mean SARA score for individuals with CA was 15.4 out of a total score of 40, and the mean SARA-gait score was 4.60 out of a total score of 8. Table 1 summarizes the participants’ baseline data.

**Table 1:** Comparison of baseline data in individuals with cerebellar ataxia and healthy Controls.

Variables	Cerebellar ataxia (*n* = 10)	Healthy control (*n* = 20)	*p*-value
Age (years) Mean ± SD	50.8 ± 9.85	48.4 ± 11.1	0.390
Gender, n (%) Male Female	9 (30.0%) 1 (3.3%)	13 (43.3%) 7 (23.3%)	0.144
Height (cm) Mean ± SD	165 ± 8.25	166 ± 5.99	0.911
SARA (/40) Mean (range)	15.4 (13.5–17.0)	-	-
SARA-gait Score (/8) Mean (range)	4.60 (4–5)	-	-

SD, Standard deviation; cm, centimeter; SARA, Scale for the Assessment and Rating for Ataxia.

*p*-value < 0.05 statistically significant.

### Gait analysis

**Fig. 2:**
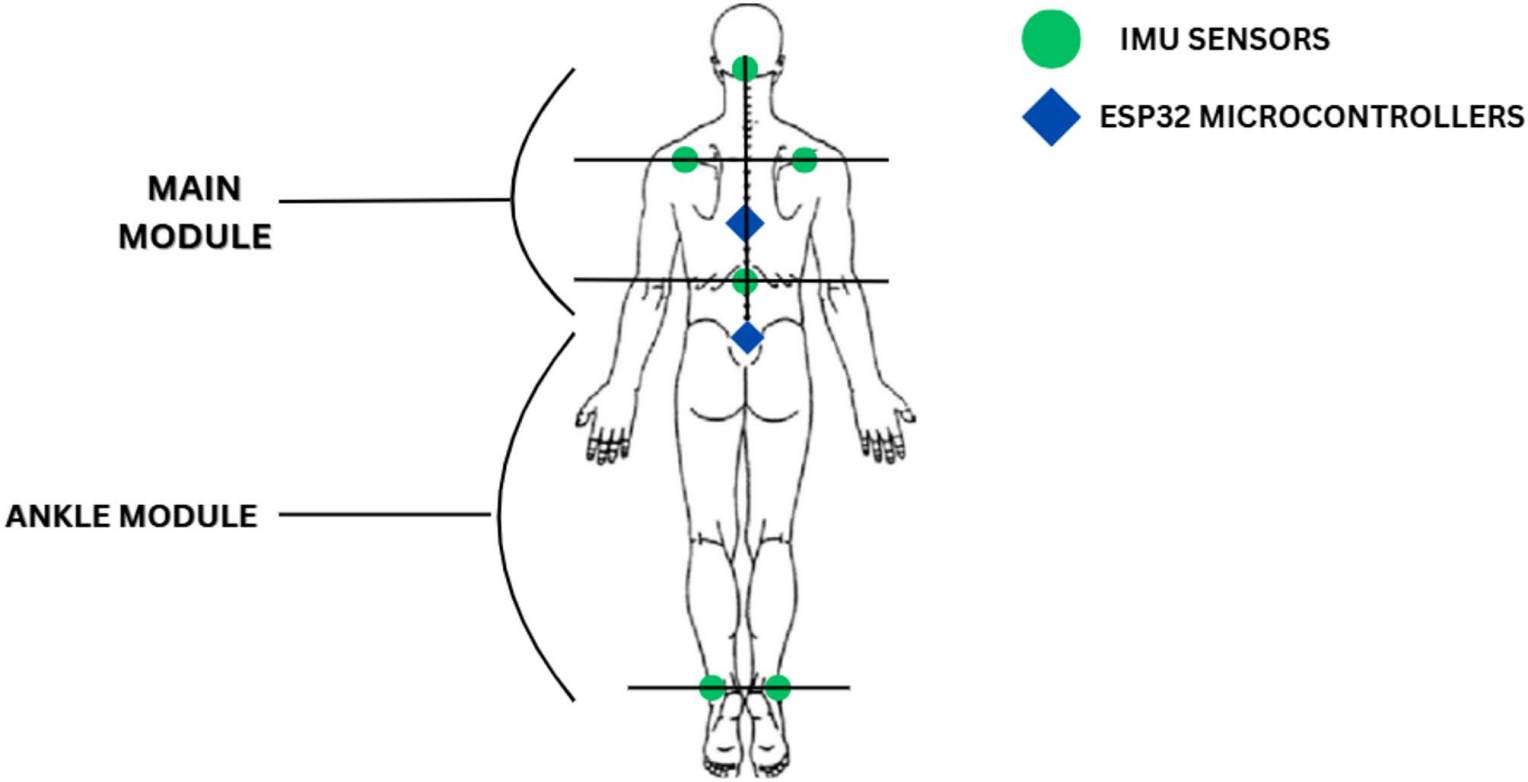
Placement of IMU sensors and Microcontrollers for trunk and gait analysis.

Kinematic data for gait analysis were collected using six MPU-9250 IMUs (16-bit/axes), which were wired with nine-axis accelerometers and gyroscope sensors. Two ESP-WROOM-32 Microcontrollers (16-bit/axes) were used, and data was stored on a laptop. The accelerometer had a measurement range of ± 2 g, and the gyroscope had a range of ± 250 degrees/sec. Additional equipment included a jacket, helmet, and Velcro straps. IMUs were positioned at strategic anatomical locations to collect kinematic data. Two sensors were mounted over the posterior aspect of each acromion process using an adjustable jacket. An additional IMU was secured on the back at the T12 vertebral level, attached to the same jacket. One sensor was fastened to the head over the external occipital protuberance via a helmet. IMU sensors were fastened to each lateral malleolus using adjustable straps. While many prior gait studies have concentrated on the lower extremities, we included trunk and upper-body placements to capture multi-segmental contributions that are often affected in cerebellar ataxia. These sites were selected based on their anatomical relevance, palpation ease for consistent placement, and feasibility in a clinical setting. Two ESP32 microcontrollers were mounted securely with the help of Velcro straps over the jacket and were utilized for wireless data acquisition. (Fig. 2). The system used Wi-Fi to collect data from the IMU sensors.

### Task description

**Fig. 3:**
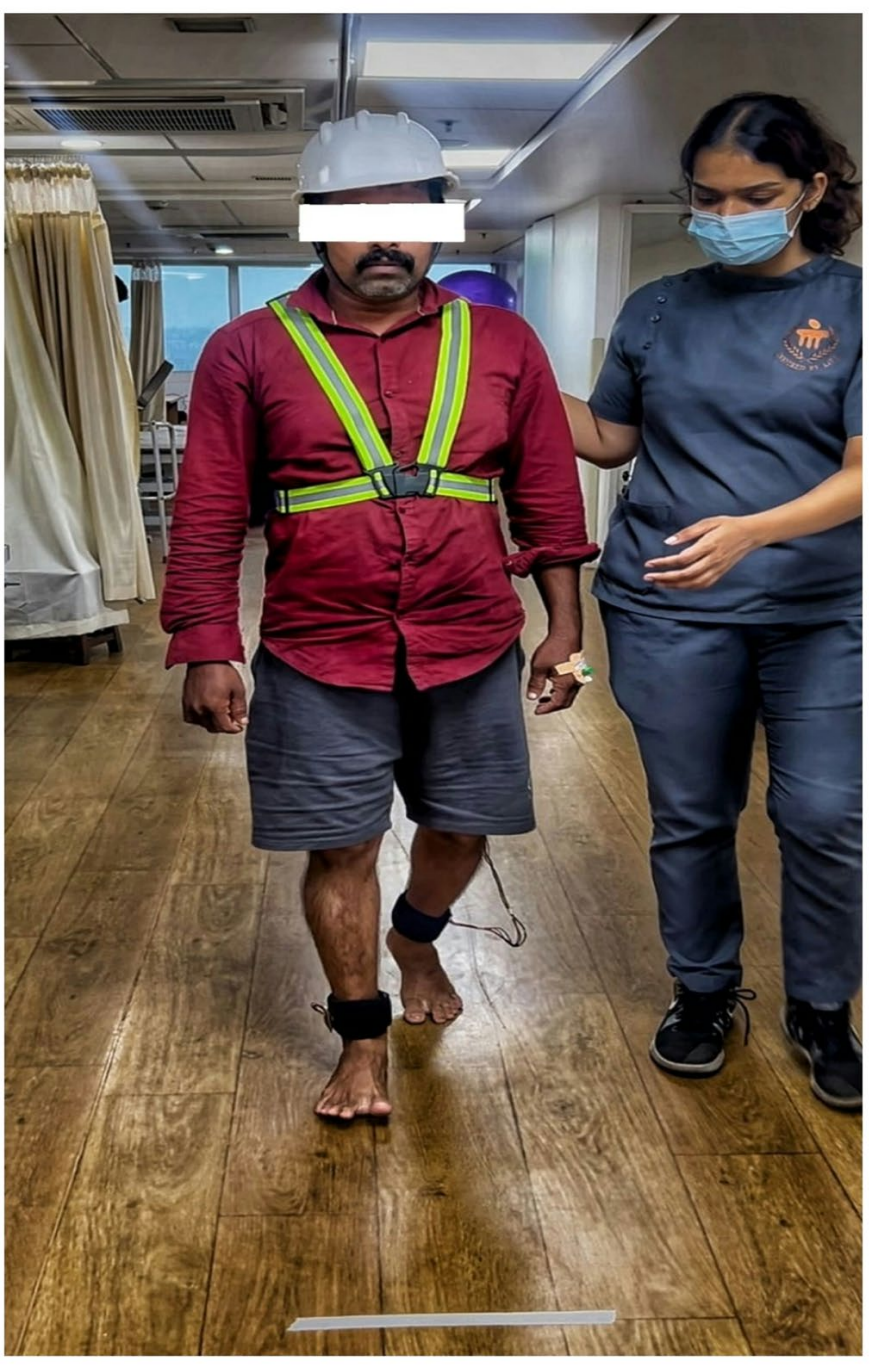
Participant performing a walking task during kinematic data collection.

Before starting the trial, to familiarize themselves with the procedure, participants performed a single walking trial along a 10-meter straight corridor (predefined path) at their own selected pace. Markings were placed at the start and end points to demarcate the distance (Fig. 3).

### Inertial sensor data processing

The IMU sensors communicated with the ESP32 microcontrollers using the Inter-Integrated Circuit protocol. Microcontroller 1 (main module) consisted of four IMUs at the head, shoulders, and lower back. Microcontroller 2 (ankle module) consisted of two IMUs at the left and right ankles.

The data were collected and sampled from the sensors at a sampling rate of 10 Hz. The ankle module gathered data from the two ankle-mounted sensors and transmitted it to the main module via Transmission Control Protocol in comma-separated values (CSV) format. Simultaneously, the main module collected data from the four IMUs (head, shoulders, and lower back), received the ankle module’s data, integrated all sensor outputs, formatted it into a CSV file for easy analysis and understanding, and sent it to the Personal Computer (PC) for real-time analysis and storage. Both the modules and the PC were connected to the same Wi-Fi network. A smartphone configured as a Wi-Fi hotspot served as the central network access point, enabling both ESP32 modules and the PC to remain connected and relay data effectively. This study used a web interface for remote data collection and control. “START” initiates sampling, while “STOP” ends it.

### Root mean square (RMS) analysis for the estimation of gait deviation

RMS values from the inertial sensor data were computed using a custom Python script to record gait variations between ataxic individuals and healthy controls. Before RMS computation, raw acceleration values from each body-worn sensor underwent a gravitational bias correction. The lower back sensor served as the postural reference, which helped estimate the gravitational acceleration. Dynamic components were isolated by estimating gravity depending on vertical axis dominance for the other sensors at the head, shoulders, and ankles. This reference helped in accurately distinguishing between the static gravitational component and the dynamic acceleration due to movement. A correlation factor was applied in the ML direction to compensate for lateral noise or minor misalignments.

RMS values were then calculated for three anatomical directions- AP, ML, and Vertical using accelerometer and gyroscope data. The RMS values expressed the degree of movement in each direction. The mean and standard deviations of the RMS values over time were taken. These aggregates provided insights into the consistency of movement, which helped differentiate between the ataxic and healthy control gait patterns. RMS was computed on the entire signal without prior segmentation. A single RMS value was calculated per sensor or per movement type, taking into account the complete time series for each axis. This approach provided a global summary of movement magnitude over the full recording duration.

RMS values were calculated using the following formulas.

The Root Mean Square (RMS) for a general signal s(*i*):

$$\:\text{RM}{\text{S}}_{\text{s}}=\sqrt{\frac{1}{N}{\sum\:}_{i=1}^{N}s{\left(i\right)}^{2}}$$

Where *s(i)* is the *i*th sample of the signal, which represents either linear acceleration [*a*_*x*_*(i)*,* a*_*y*_*(i)*,* a*_*z*_*(i)]* or angular velocity [*ωx(i)*,* ωy(i)*,* ωz(i)*]. N denotes the total number of samples.

The equation was applied separately to each axis of both accelerometer and gyroscope data to obtain RMS values for movement along each anatomical direction.

To compute the combined magnitude of motion across all three axes, the total RMS was calculated as the Euclidean norm of the individual axis RMS values:

$$\:\text{RM}{\text{S}}_{\text{total}}=\sqrt{\text{RM}{\text{S}}_{\text{x}}^{\text{2}}+\text{RM}{\text{S}}_{\text{y}}^{\text{2}}+\text{RM}{\text{S}}_{\text{z}}^{\text{2}}}$$

This total RMS computation was used for both linear acceleration and angular velocity signals. It represents the overall intensity of movement irrespective of direction.

### Angular deviation calculation

The angular deviation was computed by determining the rotational displacement between the T12 vertebral level (lower back) reference sensor and the sensor of interest through an analysis based on quaternions. A sensor fusion algorithm was utilised to obtain orientation estimates for each sensor as quaternions. Sensor orientations were corrected for axis direction mismatches.

We assumed an initial condition where the sensors were approximately aligned. All sensors were placed on the body in a consistent upright posture before data collection commenced. Due to this initial alignment, we were able to compute relative orientations with respect to the lower back sensor, which was treated as the postural reference. Implicitly, a global anatomical reference frame was established by standardised sensor placement orientations. The lower back sensor was chosen as the reference frame due to its central and relatively stable position on the body. This central reference provided an accurate estimation of the gravity vector in the initial upright posture, allowing all other sensors in the system to be aligned and corrected based on this gravitational reference.

We calculated the relative rotation between the lower back and the target sensor by taking the quaternion conjugate of the lower back orientation and multiplying it by the quaternion of the target sensor using the formula:

$$\:{q}_{\text{rel}}={q}_{\text{back}}^{*}\otimes\:{q}_{\text{sensor}}$$

Where θ denotes the angular deviation in degrees, q_rel,0_ is the scalar component of the relative quaternion. q^*^_back_ represents the conjugate of the lower back quaternion. ⊗ denotes quaternion multiplication.

The scalar component q_rel,0​_ of the resulting relative quaternion represented the cosine of half the rotation angle between the two sensor frames.

The angular deviation θ was then calculated as:

$$\:{\uptheta\:}=2\cdot\:{\text{cos}}^{-1}\left(\left|{q}_{\text{rel},0}\right|\right)$$

This formula provided the magnitude of the rotation (in degrees) required to align the target sensor’s orientation to the reference frame of the lower back.

### Statistical analysis

The kinematic data were analyzed using Jamovi software (v 2.6.44.0). Demographic characteristics of the study population were reported as means and standard deviations for continuous variables and frequencies and proportions for categorical variables. The Shapiro-Wilk test assessed whether the variables followed a normal distribution.

Kinematic variables, such as the mean RMS values from the accelerometer and gyroscope data, were compared between CA subjects and healthy individuals using the Mann-Whitney U test, as they did not follow a normal distribution. Spearman’s correlation analysis examined the relationship between the mean RMS values of accelerometer and gyroscope data and the SARA-gait score within the CA group. Furthermore, the mean angular deviation was also compared between CA subjects and healthy controls using the Mann-Whitney U test.

## Results

**Fig. 4:**
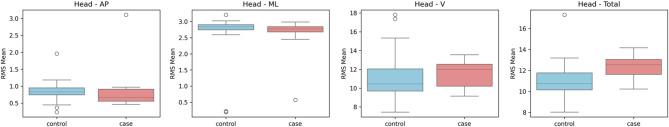
Box plots illustrating the mean RMS values of accelerometer data for the head segments in anteroposterior (AP), mediolateral (ML), vertical (V), and total directions.

**Fig. 5:**
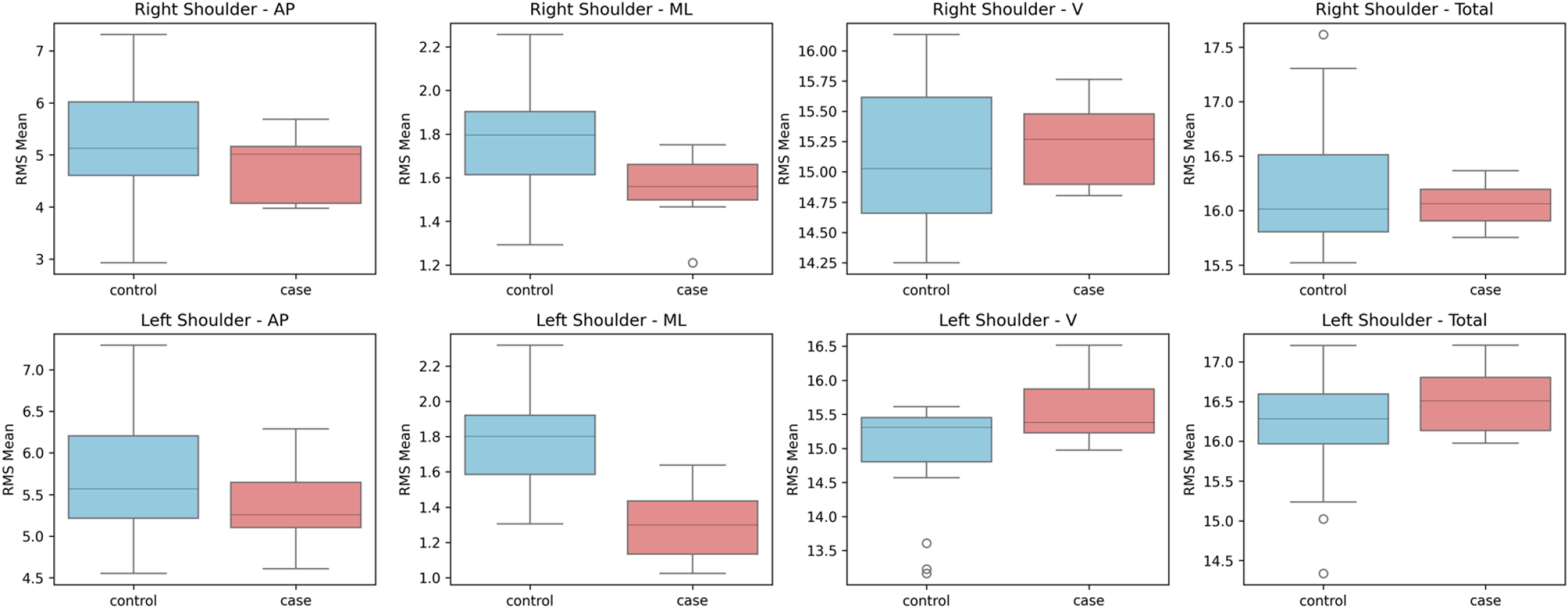
Box plots illustrating the mean RMS values of accelerometer data for the shoulder segments in anteroposterior (AP), mediolateral (ML), vertical (V), and total directions.

**Fig. 6:**
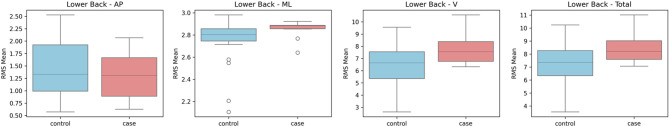
Box plots illustrating the mean RMS values of accelerometer data for the lower back segment in anteroposterior (AP), mediolateral (ML), vertical (V), and total directions.

**Fig. 7:**
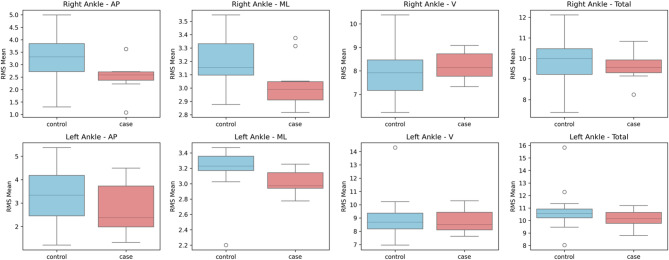
Box plots illustrating the mean RMS values of accelerometer data for the ankle segments in anteroposterior (AP), mediolateral (ML), vertical (V), and total directions.

The linear acceleration RMS median values derived from IMU accelerometers were compared between individuals with CA and healthy controls using the Mann-Whitney U test (Table 2). There were notable differences in a number of variables, mainly in the vertical and ML acceleration components (Figs. 4, 5, 6 and 7). The CA group showed a trend toward a significant decrease in the ML acceleration at the left shoulder (*p = 0.001*). In contrast, there was a substantial increase in vertical acceleration at the right ankle (*p = 0.015*), left shoulder *(p = 0.028*), and back (*p = 0.019*), indicating compensatory or erratic vertical movement patterns during locomotion.

**Table 2:** Comparison of IMU-derived accelerometer root mean square (RMS) sensor data between individuals with cerebellar ataxia and healthy Controls.

Variables†	Cerebellar ataxia Median (IQR)	Healthy Control Median (IQR)	*p*-value
Acc-head AP mean (m/s^2^)	0.0634 (0.0436–0.0862)	0.0892 (0.0768–0.1570)	0.131
Acc-head ML mean (m/s^2^)	0.2279 (0.211–0.265)	0.2657 (0.230–0.287)	0.231
Acc-head V mean (m/s^2^)	0.960 (0.956–0.972)	0.954 (0.944–0.962)	0.082
Acc-head total mean (m/s^2^)	12.3686 (11.0–13.0)	10.9335 (10.2–12.5)	0.198
Acc-R-shoulder AP mean (m/s^2^)	0.3133 (0.250–0.323)	0.3217 (0.283–0.377)	0.267
Acc-R shoulder ML mean (m/s^2^)	0.0981 (0.0931–0.105)	0.1132 (0.0995–0.119)	0.055
Acc-R shoulder V mean (m/s^2^)	0.9428 (0.939–0.962)	0.9378 (0.917–0.953)	0.061
Acc-R shoulder total mean (m/s^2^)	16.0656 (15.90–16.20)	16.0156 (15.80–16.50)	0.914
Acc-L shoulder AP mean (m/s^2^)	0.3173 (0.307–0.342)	0.3402 (0.323–0.388)	0.100
Acc-L shoulder ML mean (m/s^2^)	0.0810 (0.0677–0.0884)	0.1087 (0.0956-0.120)	0.001*
Acc-L shoulder V mean (m/s^2^)	0.9436 (0.935–0.949)	0.9318 (0.912–0.939)	0.028*
Acc-L shoulder total mean (m/s^2^)	16.5116 (16.10–16.80)	16.2856 (16.0-16.60)	0.248
Acc-back AP mean (m/s^2^)	0.1414 (0.123–0.193)	0.1743 (0.158–0.232)	0.131
Acc-back ML mean (m/s^2^)	0.3543 (0.324–0.359)	0.3868 (0.349–0.446)	0.100
Acc-back V mean (m/s^2^)	0.9175 (0.893–0.931)	0.8841 (0.852–0.911)	0.019*
Acc-back total mean (m/s^2^)	8.2004 (7.58–9.02)	7.3596 (6.33–8.26)	0.082
Acc-R ankle AP mean (m/s^2^)	0.2332 (0.219–0.268)	0.2786 (0.238–0.304)	0.155
Acc-R ankle ML mean (m/s^2^)	0.3320 (0.318–0.339)	0.3426 (0.310–0.381)	0.231
Acc-R ankle V mean (m/s^2^)	0.8783 (0.863–0.897)	0.8492 (0.810–0.867)	0.015*
Acc-R ankle total mean (m/s^2^)	9.5614 (9.31–9.96)	10.0085 (9.23–10.50)	0.619
Acc-L ankle AP mean (m/s^2^)	0.2263 (0.177–0.320)	0.2609 (0.213–0.332)	0.286
Acc-L ankle ML mean (m/s^2^)	0.3089 (0.292–0.326)	0.3200 (0.299–0.338)	0.422
Acc-L ankle V mean (m/s^2^)	0.8890 (0.848–0.919)	0.8542 (0.823–0.868)	0.109
Acc-L ankle total mean (m/s^2^)	10.1519 (9.77–10.70)	10.5557 (10.20–10.90)	0.198

†, Mann-Whitney U test; *, significant; Acc, Accelerometer data; R, Right; L, Left; AP, Anterior Posterior; ML, Mediolateral; V, Vertical; m/s^2^, meter/s^2^; IQR, Interquartile Range.

*p-value* < 0.05 statistically significant.

**Fig. 8:**
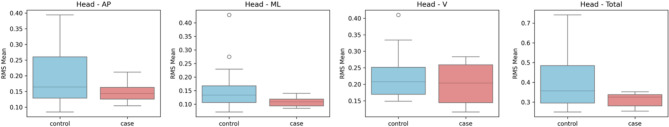
Box plots illustrating the mean RMS values of gyroscope data for the head segment in anteroposterior (AP), mediolateral (ML), vertical (V), and total directions.

**Fig. 9:**
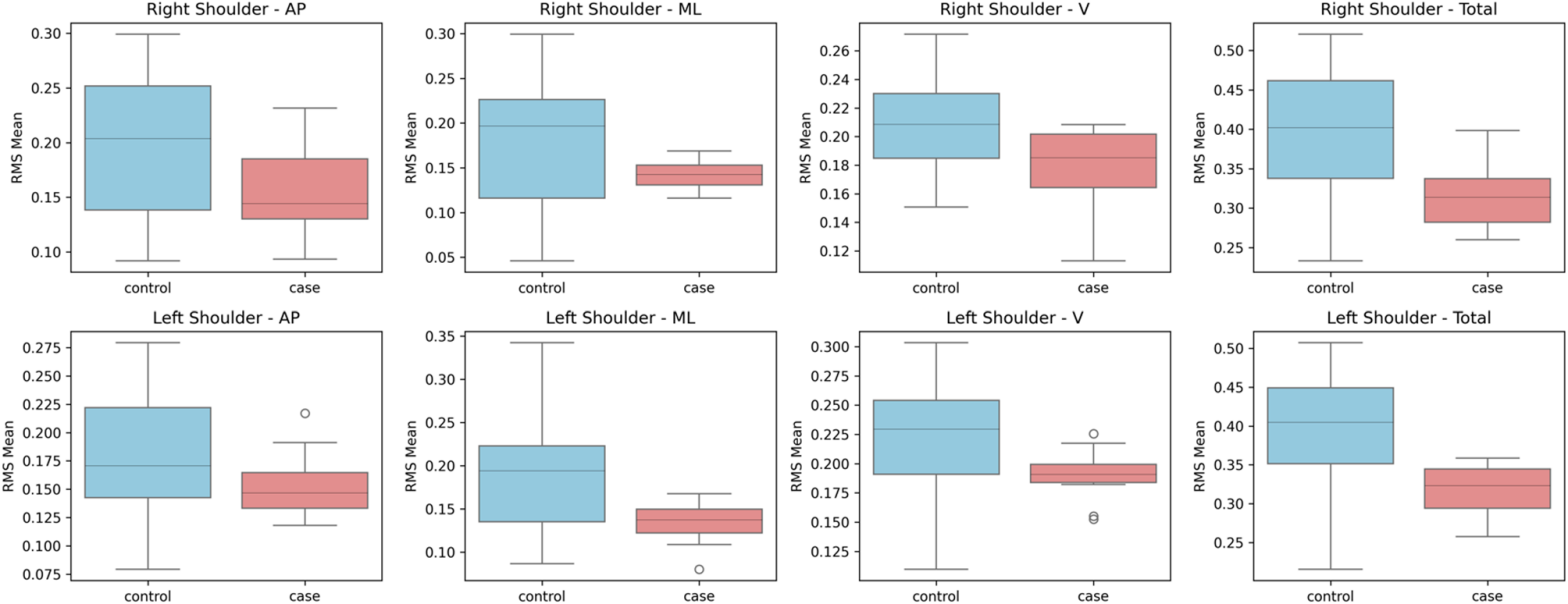
Box plots illustrating the mean RMS values of gyroscope data for the shoulder segments in anteroposterior (AP), mediolateral (ML), vertical (V), and total directions.

**Fig. 10:**
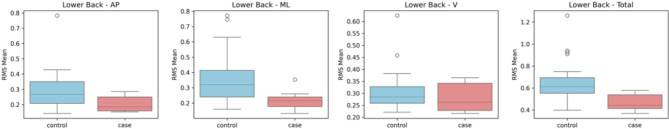
Box plots illustrating the mean RMS values of gyroscope data for the lower back segment in anteroposterior (AP), mediolateral (ML), vertical (V), and total directions.

**Fig. 11:**
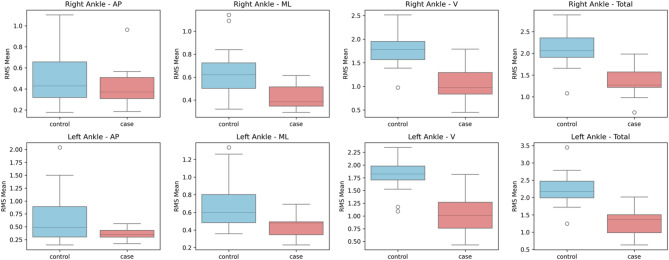
Box plots illustrating the mean RMS values of gyroscope data for the ankle segments in anteroposterior (AP), mediolateral (ML), vertical (V), and total directions.

A Mann-Whitney U test was executed on gyroscope-derived RMS data to assess angular velocity between people with CA and healthy controls (Table 3). In several body segments, noteworthy group differences were noted (Figs. 8, 9, 10 and 11). Particularly, the CA group had significantly lower total angular velocity at the right shoulder (*p = 0.017*), left shoulder *(p = 0.005)*, back *(p = 0.002)*, and both ankles (right: *p* = 0.001; left: *p* = 0.001) than healthy controls. Additionally, there was a significant reduction in the back’s vertical angular velocity (*p = 0.010*). Interestingly, individuals with CA had significantly increased AP angular velocity at the right ankle (*p = 0.013*), which may indicate compensatory or unstable gait patterns.

**Table 3:** Comparison of IMU-derived gyroscope root mean square (RMS) sensor data between individuals with cerebellar ataxia and healthy controls.

Variables†	Cerebellar ataxia Median (IQR)	Healthy control Median (IQR)	*p*-value
Gyro-head AP mean (rad/s)	0.459 (0.397–0.539)	0.468 (0.435–0.527)	0.619
Gyro-head ML mean (rad/s)	0.380 (0.320–0.426)	0.395 (0.357–0.464)	0.422
Gyro-head V mean (rad/s)	0.642 (0.571–0.789)	0.602 (0.529–0.663)	0.248
Gyro-head total mean (rad/s)	0.325 (0.280–0.338)	0.357 (0.295–0.485)	0.155
Gyro-R-shoulder AP mean (rad/s)	0.482 (0.471–0.539)	0.495 (0.447–0.542)	0.914
Gyro-R shoulder ML mean (rad/s)	0.444 (0.429–0.474)	0.461 (0.364–0.496)	0.846
Gyro-R shoulder V mean (rad/s)	0.573 (0.502–0.603)	0.542 (0.483–0.623)	0.948
Gyro-R shoulder total mean (rad/s)	0.327 (0.294–0.339)	0.402 (0.338–0.462)	0.017*
Gyro-L shoulder AP mean (rad/s)	0.481 (0.437–0.526)	0.442 (0.386–0.539)	0.373
Gyro-L shoulder ML mean (rad/s)	0.460 (0.406–0.481)	0.453 (0.400-0.532)	0.812
Gyro-L shoulder V mean (rad/s)	0.580 (0.528–0.628)	0.583 (0.530–0.644)	0.779
Gyro-L shoulder total mean (rad/s)	0.324 (0.294–0.345)	0.405 (0.351–0.449)	0.005*
Gyro-back AP mean (rad/s)	0.403 (0.382–0.421)	0.418 (0.348–0.513)	0.475
Gyro-back ML mean (rad/s)	0.451 (0.350–0.517)	0.476 (0.440–0.578)	0.214
Gyro-back V mean (rad/s)	0.068 (0.581–0.728)	0.543 (0.460–0.607)	0.010*
Gyro-back total mean (rad/s)	0.468 (0.415–0.537)	0.613 (0.552–0.693)	0.002*
Gyro R ankle AP mean (rad/s)	0.300 (0.249–0.451)	0.193 (0.175–0.293)	0.013*
Gyro-R ankle ML mean (rad/s)	0.378 (0.325–0.403)	0.329 (0.292–0.381)	0.169
Gyro-R ankle V mean (rad/s)	0.738 (0.645–0.771)	0.835 (0.798–0.885)	0.003*
Gyro-R ankle total mean (rad/s)	1.274 (1.22–1.57)	2.063 (1.91–2.36)	0.001*
Gyro-L ankle AP mean (rad/s)	0.303 (0.247–0.391)	0.202 (0.162–0.361)	0.131
Gyro-L ankle ML mean (rad/s)	0.367 (0.306–0.412)	0.301 (0.251–0.335)	0.091
Gyro-L ankle V mean (rad/s)	0.769 (0.665–0.838)	0.836 (0.751–0.869)	0.067
Gyro-L ankle total mean (rad/s)	1.362 (1.28–1.50)	2.230 (1.99–2.47)	0.001*

†, Mann-Whitney U test; *, significant; Gyro, Gyroscope data; R, Right; L, Left; AP, Anterio; Posterior; ML, Mediolateral; V, Vertical; rad/s, radians/Sect. ^2^; IQR, Interquartile Range.

*p-value* < 0.05 statistically significant.

The association between IMU-derived acceleration features and the SARA-gait score in individuals with CA was evaluated using Spearman’s rank correlation (Table 4). Overall, there were no associations that were statistically significant (all *p* > 0.05). However, a few mild, non-significant trends were found. The SARA-gait score with the right ankle’s vertical acceleration had a moderately favorable association (ρ = 0.497, *p* = 0.143), indicating that the CA group with more severe gait impairment would have higher ankle vertical acceleration. Both ankles’ ML accelerations displayed somewhat negative correlations (ρ = − 0.426, *p* = 0.219), suggesting that the degree of ataxia may be associated with an overall reduction in ML ankle dynamics during gait. Head ML acceleration demonstrated similar tendencies (ρ = − 0.355, *p* = 0.314).

**Table 4:** Spearman correlation between IMU-derived root mean square (RMS) accelerometer sensor data of individuals with cerebellar ataxia and SARA-gait score.

Variable	ρ (Spearman’s rank correlation)	*p*-value
Acc-head AP mean (m/s^2^)	0.284	0.426
Acc-head ML mean (m/s^2^)	-0.355	0.314
Acc-head V mean (m/s^2^)	-0.213	0.554
Acc-head total mean (m/s^2^)	0.355	0.314
Acc-R-shoulder AP mean (m/s^2^)	-0.071	0.845
Acc-R shoulder ML mean (m/s^2^)	0.142	0.695
Acc-R shoulder V mean (m/s^2^)	0.000	1.000
Acc-R shoulder total mean (m/s^2^)	0.142	0.695
Acc-L shoulder AP mean (m/s^2^)	0.000	1.000
Acc-L shoulder ML mean (m/s	0.284	0.426
Acc-L shoulder V mean (m/s^2^)	0.000	1.000
Acc-L shoulder total mean (m/s^2^)	0.142	0.695
Acc-back AP mean (m/s^2^)	-0.284	0.426
Acc-back ML mean (m/s^2^)	-0.213	0.554
Acc-back V mean (m/s^2^)	0.284	0.426
Acc-back total mean (m/s^2^)	0.355	0.314
Acc-R ankle AP mean (m/s^2^)	-0.284	0.426
Acc-R ankle ML mean (m/s^2^)	-0.426	0.219
Acc-R ankle V mean (m/s^2^)	0.497	0.143
Acc-R ankle total mean (m/s^2^)	0.355	0.314
Acc-L ankle AP mean (m/s^2^)	0.142	0.695
Acc-L ankle ML mean (m/s^2^)	-0.426	0.219
Acc-L ankle V mean (m/s^2^)	0.142	0.695
Acc-L ankle total mean (m/s^2^)	0.000	1.000

Acc, Accelerometer data; R, Right; L, Left; AP, Anterior-Posterior; ML, Mediolateral; V, Vertical.

*p-value* < 0.05 statistically significant.

The relationship between the SARA-gait score and angular velocity RMS readings was investigated using Spearman’s rank correlation analysis (Table 5). The SARA-gait score and the right shoulder’s ML angular velocity showed a statistically significant positive correlation (ρ = 0.640, *p* = 0.046), indicating that greater lateral rotational movement of the shoulder may be linked to more severe gait impairment in CA. No statistically significant relationships were found between other gyroscope variables. The total angular velocity at the right ankle (ρ = − 0.426, *p* = 0.219) and AP right shoulder motion (ρ = − 0.426, *p* = 0.219) displayed moderately negative trends, though, which might be indicative of compensatory mechanisms while walking in more impaired individuals with CA.

**Table 5:** Spearman correlation between IMU-derived root mean square (RMS) gyroscope sensor data of individuals with cerebellar ataxia and SARA-gait Score.

Variables	ρ (Spearman’s rank correlation)	*p*-value
Gyro-head AP mean (rad/s)	0.071	0.845
Gyro-head ML mean (rad/s)	0.000	1.000
Gyro-head V mean (rad/s)	0.000	1.000
Gyro-head total mean (rad/s)	0.284	0.426
Gyro-R-shoulder AP mean (rad/s)	-0.426	0.219
Gyro-R shoulder ML mean (rad/s)	0.0640*	0.046*
Gyro-R shoulder V mean (rad/s)	-0.142	0.695
Gyro-R shoulder total mean (rad/s)	0.000	1.000
Gyro-L shoulder AP mean (rad/s)	0.213	0.554
Gyro-L shoulder ML mean (rad/s)	0.284	0.426
Gyro-L shoulder V mean (rad/s)	-0.213	0.554
Gyro-L shoulder total mean (rad/s)	-0.071	0.845
Gyro-back AP mean (rad/s)	0.071	0.845
Gyro-back ML mean (rad/s)	-0.142	0.695
Gyro-back V mean (rad/s)	0.000	1.000
Gyro-back total mean (rad/s)	-0.142	0.695
Gyro R ankle AP mean (rad/s)	-0.213	0.554
Gyro-R ankle ML mean (rad/s)	-0.142	0.695
Gyro-R ankle V mean (rad/s)	0.142	0.695
Gyro-R ankle total mean (rad/s)	-0.426	0.219
Gyro-L ankle AP mean (rad/s)	0.213	0.552
Gyro-L ankle ML mean (rad/s)	-0.071	0.845
Gyro-L ankle V mean (rad/s)	0.000	1.000
Gyro-L ankle total mean (rad/s)	-0.284	0.426

*, Significant; Gyro, Gyroscope data; R, Right; L, Left; AP, Anterior-Posterior; ML, Mediolateral; V, Vertical.

*p-value* < 0.05 statistically significant.

Each variable is the mean angular deviation averaged across all time points between the named sensor and a fixed back‑mounted reference sensor, expressed in degrees (Table 6). Medians were reported because the distributions were skewed, and group differences were tested with Mann–Whitney U tests. There were no apparent differences at the right shoulder (*p = 0.267*) or head (*p = 0.248*). Although it did not achieve statistical significance, the left shoulder revealed a trend toward decreased angular deviation in the ataxia group (median 3.189° vs. 3.750°, *p = 0.067).* Both ankle measurements, however, were significantly lower in the CA group: the median displacement of the right ankle was − 0.457° (1.147° vs. 1.604°, *p* = 0.001), and the median displacement of the left ankle was − 0.313° (1.209° vs. 1.522°, *p = 0.006).* These findings show that CA subjects with limb ataxia maintained proximal control.

**Table 6:** Comparison of angular deviation between individuals with cerebellar ataxia and healthy Controls.

Variables†	Cerebellar ataxia (median)	Healthy control (median)	*p*-value
Angular deviation head-mean (°)	3.265	3.703	0.248
Angular deviation R shoulder mean (°)	3.285	3.614	0.267
Angular deviation L shoulder mean (°)	3.189	3.750	0.067
Angular deviation R ankle mean (°)	1.147	1.604	0.001*
Angular deviation L ankle mean (°)	1.209	1.522	0.006*

†, Mann-Whitney U test; *, significant; R, Right; L, Left; °, degrees.

*p*-value < 0.05 statistically significant.

## Discussion

This novel study investigated trunk kinematics and gait in individuals with CA (*n* = 10) and healthy participants (*n* = 20) using IMUs. Notably, along the shoulders and back segments, our study found that in individuals with CA, RMS angular and RMS linear accelerations were lower than those of healthy controls. These alterations reveal a dysfunction in the trunk’s ability to control gait dynamically. In the past, gait variability has been explored using sensors usually placed on the lower body segments^20–26^. By capturing multi-axis kinematic segmental RMS data from the head, shoulders, lower back, and ankles, our study contributes to the existing evidence by highlighting the key characteristics distinguishing normal gait dynamics from abnormal motor control.

In healthy individuals, accurate and efficient movement relies on internal models within the brain that integrate sensory input with motor commands to predict and adapt to changes in limb position and velocity. Based on specific theories, the cerebellum might have two kinds of internal models: an inverse model that can compute the appropriate motor command for a desired change in state, position, and velocity, and a forward model that can predict and anticipate the next state based on the current state and motor command. Cerebellar damage, as seen in CA, disrupts these internal models, leading to motor deficits such as dysmetria and dyssynergia^4,29^. CA individuals exhibit slower, less coordinated movements with increased variability, particularly in multi-joint and inter-limb coordination. Gait is often characterized by reduced velocity, prolonged movement duration, and poor dynamic balance due to inaccurate foot placement and impaired limb coordination^29^.

We observed a significant decrease in ML RMS linear acceleration at the left shoulder (*p* = 0.001), which aligns with previous studies. An adaptive mechanism to lessen the lateral sway and stabilize the upper body may be reflected in this data. Decreased gait speeds are commonly noticed in CA individuals^6^. While Caliandro et al. addressed poor upper-lower segment coordination as a contributing factor to this reduction^28^, Sekine et al. revealed a robust association between RMS acceleration and walking speed^30^. Individuals with CA may tighten their upper limbs and the trunk without precise feedforward control to maintain balance during walking.

Compensatory or irregular vertical motion can be one of the probable reasons for increased vertical RMS linear acceleration at the left shoulder (*p = 0.028*), right ankle (*p* = 0.015), and back (*p* = 0.019). These results are consistent with research done by Conte et al., showing that neck/trunk hypotonia and poor pelvis-trunk coordination cause higher trunk oscillations^11^. Vertical variations can be caused by abnormal push-off and heel-strike dynamics, abnormal foot alignment, and abnormal lower limb kinematics. Therefore, the downstream effects of cerebellar impairment on vertical postural control are reflected in our data.

Total RMS angular velocity was found to be considerably lower at the back (*p* = 0.002), left shoulder (*p* = 0.005), right shoulder (*p* = 0.017), and both ankles (*p* = 0.001). These findings show that individuals with CA use joint stiffening techniques through greater muscle coactivation to compensate for hypotonia and poor postural control, as stated by Manto et al.^4^. AP RMS angular velocity (*p* = 0.013) increased substantially at the right ankle. When proximal segments stiffen (rigid), this can indicate a compensatory push-off technique. Smaller and slower ankle sub-movements were reported in CA individuals by Ilg et al.^31^; nevertheless, our data suggests that CA individuals may compensate with distal muscle activation when proximal control is poor.

Correlation analysis showed no statistically significant associations (*p* > 0.05) between the IMU-derived accelerometer RMS mean values and the SARA-gait score of the CA group. Greater lateral rotational movement of the shoulder may be linked to more severe gait impairment in CA, as demonstrated by a statistically significant positive relationship between the SARA-gait score and IMU-derived gyroscope RMS values at the right shoulder ML RMS value (ρ = 0.640, *p* = 0.046). Comparison of angular deviation between CA and healthy controls showed that both ankle measurements were notably lower in the CA group; the median displacement of the right ankle was − 0.457° (*p = 0.001*), and the median displacement of the left ankle was − 0.313° (*p = 0.006*). These findings align with the study done by Pau et al., who found that ankle dorsiflexion and plantarflexion angles were reduced in the CA group during terminal stance and pre-swing phases^32^.

These findings point to a structural breakdown in inter-limb and intra-limb coordination. A dissociation in upper-lower segment coupling is reflected in reduced angular movement in proximal segments and increased distal effort^28^. These results correspond with symptoms of CA, such as dyssynergia and dysmetria^8^.

It is fundamental to accept several methodological limitations in this study, even though this research offers notable insights into the trunk kinematics and gait in people with CA and healthy controls. Firstly, as a pilot study, the small sample size limited the findings’ statistical power, and an unbalanced gender distribution, with a predominance of male participants, affected generalizability. It also hindered the possibility of conducting a subgroup analysis of the participants based on the presence or absence of truncal ataxia. Such important considerations could offer more comprehensive information about gait patterns in this cohort. Secondly, A sampling frequency of 10 Hz was used for data acquisition to ensure technical feasibility and clinical applicability. This frequency may not have effectively detected subtle or rapid postural modifications, even though it appeared sufficient to record large trunk movements while walking. The selection of sampling frequency was constrained due to the simultaneous use of two microcontrollers, six sensors, and data handling limitations. The limited correlation with SARA-gait score may reflect the differences between objective IMU-derived measures and clinician-rated ordinal scales, compounded by the small sample size and the exploratory nature of this pilot study. Although sensor-to-segment calibration and gait speed normalization were not performed, these choices were intentional to prioritize feasibility within a real-world hospital setting. Our approach emphasized ecological validity by allowing self-selected walking speeds and using straightforward anatomical alignment without additional calibration, making the methodology more adaptable to routine hospital practice. Logistical challenges like time constraints of the study and difficulties in recruiting individuals with CA with clinically evident truncal ataxia further restricted the scope of this research.

This study evaluated the head and trunk kinematics and gait and their variability in CA individuals and compared the differences with healthy individuals using wearable IMU sensors. The findings underscore the need for analyzing the trunk and gait kinematics using wearable IMU sensors, as they offer an objective method that bridges the gap between subjective scales and expensive, laboratory-based objective motion capture analysis in neurorehabilitation practice. These findings may provide an objective assessment, enhance clinical decision-making, and help design targeted rehabilitation treatments focusing on trunk control or developing innovative orthotic devices to reduce trunk oscillations and potentially optimize dynamic balance in ataxic individuals, reducing their fall risk and improving their quality of life.

Future studies should recruit more samples, including subgroups with or without truncal ataxia, and use power estimation for a more robust statistical analysis. Sensors at a higher sampling frequency should be employed to detect subtle and rapid changes that would provide a more comprehensive assessment of the trunk kinematics during gait. Including gait speed measurement with normalization and extending analyses beyond RMS to encompass temporal and asymmetry parameters would allow a more comprehensive characterization of ataxic gait. Incorporating EMG with IMU-based gait analysis would provide deeper insights into muscle activation patterns and help differentiate between compensatory strategies and abnormal motor patterns.

## Conclusions

The study’s findings revealed an overall reduced RMS linear and angular velocity in the CA group. Wearable IMU sensors should be employed for recording trunk kinematics during gait, a fundamental component of analysis in individuals with CA. The results pave the way to more focused and objective assessment by validating the beneficial role of trunk-focused evaluation in comprehending balance and postural control during gait in individuals with CA.

## Data Availability

The data presented in this study are openly available at (https:/doi.org/10.17605/OSF.IO/M9YV8).

## Abbreviations

AP: Anterior–posterior
CA: Cerebellar ataxia
CSV: Comma-separated values
IMU: Inertial measurement units
ML: Mediolateral
PC: Personal computer
RMS: Root mean square
SARA: Scale for the assessment and rating of ataxia
V: Vertical
